# Identification and Functional Characterization of the FATP1 Gene from Mud Crab, *Scylla paramamosain*

**DOI:** 10.3390/ani14202969

**Published:** 2024-10-15

**Authors:** Wenjie Zhong, Chuangsi Chen, Senyue Tan, Xianda He, Xiaobo Wen, Shuqi Wang, Douglas R. Tocher, Khor Waiho, Cuiying Chen

**Affiliations:** 1 Guangdong Provincial Key Laboratory of Marine Biotechnology, Institute of Marine Sciences, Shantou University, Shantou 515063, China; 22wjzhong1@stu2022.jnu.edu.cn (W.Z.); 22320221151374@stu.xmu.edu.cn (C.C.); 21sytan@stu.edu.cn (S.T.); xdhe@stu.scau.edu.cn (X.H.); sqw@stu.edu.cn (S.W.); d.r.tocher@stir.ac.uk (D.R.T.); 2 College of Marine Sciences, South China Agricultural University, Guangzhou 510642, China; wenxbo@scau.edu.cn; 3 Institute of Aquaculture, Faculty of Natural Sciences, University of Stirling, Stirling FK9 4LA, UK; 4 Higher Institution Centre of Excellence (HICoE), Institute of Tropical Aquaculture and Fisheries, University Malaysia Terengganu, Kuala Terengganu 21300, Malaysia; waiho@umt.edu.my

**Keywords:** *Scylla paramamosain*, FATP1, clone, lipid accumulation

## Abstract

**Simple Summary:**

The role of fatty acid transport protein 1 (FATP1) in decapod crustaceans is still poorly understood. In this study, we cloned the FATP1 cDNA from the mud crab *Scylla paramamosain*, identified its sequence features and tissue distribution pattern and analyzed its roles in lipid metabolism in vivo. We found that FATP1 is involved in long-chain polyunsaturated fatty acid metabolism and deposition in crustaceans, which provides new information for understanding the function of FATP1 in invertebrates.

**Abstract:**

In mammals, fatty acid transport protein 1 (FATP1) plays important roles in cellular uptake and activation of long-chain fatty acid (LCFA), especially in processes of transportation, oxidation and triacylglycerol synthesis. However, the role of FATP1 in invertebrates, especially decapod crustaceans, is still poorly understood. In this study, the cDNA of a FATP1 gene from a decapod crustacean, mud crab *Scylla paramamosain*, was cloned and functionally characterized. The FATP1 gene encoded a polypeptide consisting of 643 amino acids that exhibits all the typical features of the FATP family and shares high homology with the other FATP orthologs of crustaceans. The relative mRNA expression levels of *FATP1* were observed to be higher in metabolically active tissues such as hepatopancreas, stomach and gill than in other crab parts. Knockdown of the *FATP1* mRNA in vivo significantly reduced triacylglycerols and total lipid levels in the hepatopancreas, accompanied by an increase in the expression of genes related to fatty acid transportation, allocation and hydrolysis, including long-chain acyl-CoA synthetase 3/4 (*ACSL3/4*) and carnitine palmitoyl transferase 1 (*CPT1*), and a decrease in the expression of genes related to fatty acid synthesis such as acetyl-CoA carboxylase (*ACC*) and fatty acid synthase (*FAS*) in the hepatopancreas. Furthermore, increased dietary n-3 long-chain polyunsaturated fatty acid (LC-PUFA) levels resulted in the up-regulation of the *FATP1* expression in the hepatopancreas, accompanied by an increase in LC-PUFA content, especially eicosapentaenoic acid (EPA, 20:5n-3) and docosahexaenoic acid (DHA, 22:6n-3), in both polar (PLs) and neutral lipids (NLs) in the hepatopancreas and muscles of crabs. These findings suggested that the FATP1 gene identified in *S. paramamosain* might play important roles in regulating long-chain fatty acid metabolism and deposition in crustaceans.

## 1. Introduction

Fatty acid transporter proteins (FATPs), also known as solute carrier family 27, are a family of proteins involved in the processes of cellular uptake and activation of long-chain fatty acids (LCFAs, fatty acids with a carbon chain length greater than 12 carbon atoms) [1,2]. So far in mammalian genomes, six proteins have been identified as members of the FATP family, designated as FATP1–6, all of which are integral membrane proteins with a molecular weight of 63–80 kDa, an N-terminus located extracellularly, a C-terminus intracellularly and at least one transmembrane domain [2,3,4]. In addition, all FATP members have a highly conserved sequence named the FATP sequence, also known as the AMP binding domain, on the C-terminus [2]. It consists of about 311 amino acids and is important for FATP-mediated fatty acid (FA) transport [5,6]. Although the sequences of FATPs are similar, the expressed proteins exhibit highly distinct patterns of tissue distribution and each plays a discrete role in maintaining lipid homeostasis associated with FA uptake [7,8].

FATP1, the first member of the FATP family to be identified through the screening of a cDNA library from 3T3-L1 adipocytes for LCFA uptake, showed a preference for a broad range of FAs and was highly expressed in tissues with rapid FA metabolism, such as the heart, skeletal muscle, liver and adipose tissue [9,10,11]. Previous studies revealed that human FATP1 protein has 646 amino acids and contains the transmembrane domain, an AMP binding motif and multiple membrane-associated domains peripherally associated with the inner leaflet of the plasma membrane [4,9,12]. Gain- and loss-of-function studies have demonstrated that FATP1 is vital for cellular LCFA uptake and activation and involved in the processes of LCFA transportation and oxidation, triacylglycerol synthesis, lipid accumulation and adipocyte differentiation [2,13]. Knockdown of *FATP1* significantly reduced triacylglycerol accumulation and lipid droplet sizes in 3T3-L1 adipocytes [14] and LCFA uptake and lipid accumulation in adipocytes and skeletal muscle of mice [15,16]. Similarly, using gain-of-function analysis, both LCFA uptake and lipid accumulation in adipocytes, heart and skeletal muscle were found to be facilitated by overexpression of *FATP1* [17]. However, other studies reported that overexpression of *FATP1* did not increase lipid accumulation in rat L6E6 myotubes [18] and that lipid accumulation in QM-7 (quail muscle) cells and mouse skeletal muscle was inhibited by overexpression of *FATP1* [19,20]. While it was argued whether FATP1 can promote or inhibit FA uptake and lipid accumulation in different cell types or tissues in mammals, further studies are clearly required to characterize and validate the function of FATP1 in other nonmammalian animal species. 

Although the precise mechanism is still not fully understood, mammal FATP1 has been shown to play a vital role in the uptake and activation of LCFA in vertebrates [3,13]. Several FATPs or orthologs have also been identified and characterized in invertebrates. In wild-type *Drosophila*, FATP is required for the expansion of lipid droplets, which are indispensable for photoreceptor survival during aging in retinal pigment cells [21]. In silk moth *Bombyx mori*, FATP was shown to facilitate lipid droplet accumulation and triacylglycerol synthesis within pheromone gland cells [22]. In *Caenorhabditis elegans*, mutants of two genes, ACS-20 and ACS-22, homologous to mammalian FATP4, reduced the incorporation of exogenous very-long-chain fatty acids (VLCFAs, fatty acids with a chain length of 22 carbon atoms or more) into sphingomyelin, which is associated with severely disrupted cuticle surface barrier function [23]. Moreover, several studies have found that the mRNA expression levels of *FATP* in decapod crustaceans were affected by dietary FA levels [24,25,26]. However, until now, the functions of FATPs involved in LCFA transport and activation in crustaceans had not been fully verified. 

Mud crab (*Scylla paramamosain*), renowned for its large size, delicious meat and high nutritional and economic value, is one of the most valuable crustaceans and cultured widely in the Indo-West Pacific region [27,28]. The nutritional value of mud crab is enhanced by their high lipid content abundant in edible tissues such as the hepatopancreas, ovary and muscle, especially the presence of omega-3 long-chain polyunsaturated fatty acids (n-3 LC-PUFAs, fatty acids with a carbon chain length ≥ 20 carbon atoms, two or more double bonds and the first double bond located in the third carbon atom from the methyl terminus), eicosapentaenoic acid (EPA, 20:5n-3) and docosahexaenoic acid (DHA, 22:6n-3) [27,29]. Given the widely studied involvement of FATP1 in lipid metabolism in vertebrates, we cloned and identified the FATP1 gene cDNA from an invertebrate, *Scylla paramamosain*, and analyzed its functional roles in lipid metabolism and deposition, with the expectation of providing new information to illuminate how FATP1 is involved in absorption, utilization and allocation of dietary lipids in crustaceans. 

## 2. Materials and Methods

### 2.1. Experimental Animals and Sampling

All operations performed on crabs were in accordance with the National Institutes of Health Guide for the Care and Use of Laboratory Animals (NIH Publications No. 8023, revised 1978) and approved by the Institutional Animal Care and Use Committee of Shantou University (No. 132, revised 6 July 2022; Guangdong, China). 

Juvenile mud crabs (*S. paramamosain*) used in the feeding trial and RNA interference experiment were collected from the estuary of the Rongjiang River in Shantou City, China. Before the experiment, the crabs were placed into individual polypropylene tanks (32 cm × 20 cm × 14 cm) of an indoor recirculating aquaculture system (Zhongkehai, Qingdao, China) and acclimated to brackish water (15 g/L) with commercial feed for two weeks. 

In addition, six healthy adult male mud crabs (body weight of 210.79 ± 10.58 g) with stage III testis development were obtained from the Niutianyang coastal area of Shantou City (Guangdong Province, China) and used for the acquisition of different tissues. These crabs were cultured artificially by feeding Chinese mystery snail *Cipangopaludina chinensis* and small saltwater clam *Potamocorbula rubromuscula* in offshore ponds, where the salinity was 5.5 to 10.2 ppt, ambient water temperature 20.0 to 30.0 °C, pH 7.4 to 8.1, dissolved oxygen between 4.0 and 7.8 ppm and ammonia–nitrogen < 0.05 ppm. Several tissues including hemocyte, eyestalk, heart, intestine, muscle, gill, ganglion, stomach, hepatopancreas and gonad were dissected rapidly and frozen immediately in liquid nitrogen as described previously [30]. All tissues samples were then stored at −80 °C until RNA extraction.

### 2.2. Feeding Trial

After acclimated in individual polypropylene tanks (32 cm × 20 cm × 14 cm) of an indoor recirculating aquaculture system for two weeks, ninety healthy crabs (initial average weight, 56.66 ± 1.57 g) were allocated randomly into five dietary treatments with eighteen crabs in each treatment. Crabs were fed with 5 isoproteic (41.7% crude protein) and isolipidic (7.8% crude lipid) diets containing graded levels (0.50, 0.73, 1.04, 1.42 and 2.57% of dry weight, respectively) of n-3 LC-PUFA, predominantly EPA and DHA, with a constant level (0.52% of dry weight) of n-6 LC-PUFA (fatty acids with a carbon chain length ≥ 20 carbon atoms, two or more double bonds and the first double bond located in the sixth carbon atom from the methyl terminus), mainly arachidonic acid (ARA, 20:4n-6). The experimental diets were prepared and stored as described in detail previously [31], and formulations and analyzed proximate compositions are shown in Supplemental Table S1. 

During the feeding trial, crabs were fed to apparent satiety twice a day at 7:00 and 18:00, the water temperature ranged between 25 °C and 28 °C, pH between 7.4 and 8.0, dissolved oxygen from 5.0–7.0 mg/L, ammonia nitrogen < 0.01 mg/L and salinity was about 15 g/L. After the 9-week feeding trial, the crabs were starved for 24 h and chilled on ice to anesthetize, and then the hepatopancreas and muscle from 5 crabs per treatment were collected and stored at −80 °C for subsequent fatty acid composition and gene expression analysis.

### 2.3. Cloning and Sequencing of S. paramamosain FATP1 cDNA

Total RNA was isolated by RNA isolator reagent according to the manufacturer’s protocol (Vazyme Biotech Co., Nanjing, China). The integrity, concentration and quality of total RNA were detected using a NanoDrop One spectrophotometer (Thermo Scientific, Waltham, MA, USA) and 1.0% agarose gel electrophoresis, respectively. The first-strand cDNA was synthesized using HiScript III RT SuperMix for qPCR (+gDNA wiper) reagent under the manufacturer’s instructions (Vazyme Biotech, Nanjing, China). 

The full-length cDNA of *FATP1* gene was amplified based on the transcriptome database of *S. paramamosain* [32] with HiScript-TS 5′/3′ RACE Kit following the manufacturer’s protocol (Vazyme Biotech, Nanjing, China). The used specific primers are shown in Supplementary Table S2. The PCR products were purified and cloned into pCE2 TA/Blunt-Zero Vector (Vazyme Biotech, Nanjing, China), which was then transformed into competent cells *E. coli* DH5α as described previously [30]. The positive clones were identified by PCR and then selected to be sequenced commercially by Sangon Biotech Co., Ltd. (Shanghai, China).

### 2.4. Bioinformatic Analysis

The complete open reading frame (ORF) and amino acid sequences of the FATP1 gene of mud crab was obtained by DNAMAN V9.0 based on the sequencing results. Then, the biochemical properties of the encoding protein were analyzed using the ProtParam online tool (http://web.expasy.org/protparam/, accessed on 22 April 2022). We compared the protein sequences of FATP1 orthologs from vertebrates and invertebrates with that of *S. paramamosain* using multiple sequence alignment by ClustalW (https://www.genome.jp/tools-bin/clustalw, accessed on 30 October 2023) and constructed the phylogenic analysis by MEGA software V6.0.

### 2.5. RNA Interference

To synthesize double-stranded RNA (dsRNA) for knocking down the expression of the *FATP1* gene in *Scylla paramamosain*, a prokaryotic expression vector was constructed as described in detail previously [30]. Briefly, a 438 bp fragment of *FATP1* full-length cDNA was chosen and amplified by PCR using specific primers (Supplementary Table S2) to produce the corresponding dsRNA according to its sequence characteristics. After purification, the PCR product was digested with *EcoR* I and *Stu* I and then ligated into the pLITMUS-28i vector (NEB, Ipswich, MA, USA), followed by transformation into *E. coli* HT115 (DE3) competent cells. The single positive clone, verified by restriction enzyme digestion and sequencing, was inoculated in LB medium containing 10 μg/mL ampicillin and tetracycline (Sigma-Aldrich, St. Louis, MO, USA) at 37 °C with shaking at 250 rpm/min for 4 h (OD600 ≈ 0.4–0.6). Then, 0.1 mM/L isopropyl-β-d-thiogalactoside (IPTG) was added and the mixture was cultured for another 4 h to induce the expression of single-stranded RNA. Subsequently, the dsRNA was synthesized by annealing at 70 °C for 10 min after total RNA extraction. The redundant single-strand RNA and DNA were then removed by RNase A and DNase I digestion, respectively (Sangon Biotech, Shanghai, China). After confirming the concentration, quality and reliability, all dsRNA was stored at −80 °C until further use.

Before the RNA interference experiment, we verified the effective injection concentration and interval time of the dsRNA in mud crab. After acclimating in breeding tanks for two weeks, 48 healthy crabs in total with an average weight of 5 g were randomly selected and equally divided into two treatments, with 24 individuals per treatment. One treatment was injected with dsRNA (3 mg/kg body weight) diluted in sterilized phosphate buffered saline (PBS) into the arthropodial membrane at the base of the walking legs every 3 days for 2 weeks, while another group as the negative control was treated with an equal volume of PBS. During the experiment, crabs were fed the diet with 1.04% n-3 LC-PUFA (see Supplementary Table S1) and cultured in the conditions of water temperature at 25–28 °C, pH at 7.4 to 8.0, ammonia nitrogen < 0.01 mg/L, dissolved oxygen > 5.0 mg/L and salinity of about 15 g/L.

At the end of the RNA interference experiment, all surviving crabs were fasted for 24 h before dissection. Crabs were chilled on ice for general anesthesia, and hemolymph, hepatopancreas and muscle were collected. Due to the low weight of the tissues sampled from 1 crab, the hemolymph, hepatopancreas and muscle of 4–6 crabs were pooled into 1 replicate, and each treatment had 4 replicates. The pooled samples were then stored at −80 °C for subsequent biochemical, FA composition and gene expression analysis. Additionally, a part of the hepatopancreas from three crabs per treatment was collected and stored in 4% paraformaldehyde (Biosharp, Hefei, China) for histological analysis.

### 2.6. Tissue Total Lipid, Polar Lipids (PLs), Neutral Lipids (NLs), Triacylglycerol (TG) and Cholesterol (T-CHO) Contents and FA Composition Analysis

TG and T-CHO contents in the hepatopancreas of *S. paramamosain* were determined by commercial assay kits according to the manufacturer’s protocols (Nanjing Jiancheng Bioengineering Institute, Nanjing, China). Total lipids of hepatopancreas and muscle were extracted with a mixture of chloroform/methanol (2:1, by vol.) containing 0.01% butylated hydroxytoluene as antioxidant according to the method described by Folch et al. [33]. The total lipids of hepatopancreas and muscle collected from the feeding trial were then fractionated into NLs and PLs by chromatography using Sep-Pak@ Vac 6 cc (500 mg, 30/pk) silica cartridges (Waters, Milford, MA, USA). The NLs and PLs were eluted with chloroform and methanol, respectively, as described by Belaunzaran et al. [34], and the FA methyl of these lipids was prepared as follows. Lipids were saponified using 0.5 M potassium hydroxide in methanol before being subjected to methylation by boron trifluoride methanol (ca. 14%; Acros Organics, Newark, NJ, USA). FA methyl esters were then separated by gas chromatography (GC-2010 plus, Shimadzu, Kyoto, Japan) equipped with an auto-sampler and a hydrogen flame ionization detector as previously described in detail by Li et al. [35]. Individual FAs were identified using the known commercial standards (Sigma-Aldrich, St. Louis, MO, USA) and quantified with the percentage of each FA calculated using the peak area normalization method in a GC solution workstation (Shimadzu, Kyoto, Japan).

### 2.7. Histological Analysis

Histological analysis was conducted as described in detail previously [36]. Briefly, the samples stored in 4% paraformaldehyde were frozen, fixed and sectioned (5 μm) and then stained with Oil Red O (ORO). A light microscope (Axio Imager 2, Zeiss, Oberkochen, Germany) equipped with a camera (Axiocam 506, Zeiss) and software (ZEN 2, Zeiss) was used for image acquisition of sections. Image-pro plus version 6.0 (Media Cybernetics, Rockville, MD, USA) was used to quantify the lipid droplets stained by ORO.

### 2.8. Quantitative PCR (qPCR) Analysis

The mRNA expression levels of genes including fatty acid transport protein 1/4 (*FATP1/4*), long-chain acyl-CoA synthetase 1/3/4 (*ACSL1/3/4*), acetyl CoA carboxylase (*ACC*), fatty acid synthetase (*FAS*), sterol regulatory element binding protein 1 (*SREBP1*), hormone-sensitive lipase (*HSL*) and carnitine palmitoyltransferase 1 (*CPT1*) in hepatopancreas and muscle tissues were detected by SYBR-Green-based real-time qPCR. 

Total RNA was extracted as described above and 1 μg of high-quality RNA was reverse-transcribed using a HiScript III RT SuperMix for qPCR (+gDNA wiper) kit (Vazyme Biotech, China). All qPCR assays were performed in a LightCycler^®^480 thermocycler (Roche, Basel, Switzerland) as described previously [30]. Each sample was run in triplicate, and reactions without templates were used as negative controls. The *18s rRNA* gene was used as the internal control gene to normalize the experimental data. The relative transcript levels of each gene were calculated by the 2^−ΔΔCt^ method. Primers used in qPCR are shown in Supplementary Table S2.

### 2.9. Statistical Analysis

After checking for normal distribution and homogeneity of variance of the data by the Kolmogorov–Smirnov and Barlett’s tests, an independent sample *t*-test for two groups or one-way ANOVA and Tukey’s test for multiple groups at the *p* < 0.05 level of significance were performed with SPSS software (version 26.0, IBM, Armonk, NY, USA). Results were expressed as means ± standard error of the mean (SEM). Origin 2017 (OriginLab Corporation, Northampton, MA, USA) was utilized for the preparation of all graphical representations.

## 3. Results

### 3.1. Sequence and Phylogenetic Analysis

**Figure 1:**
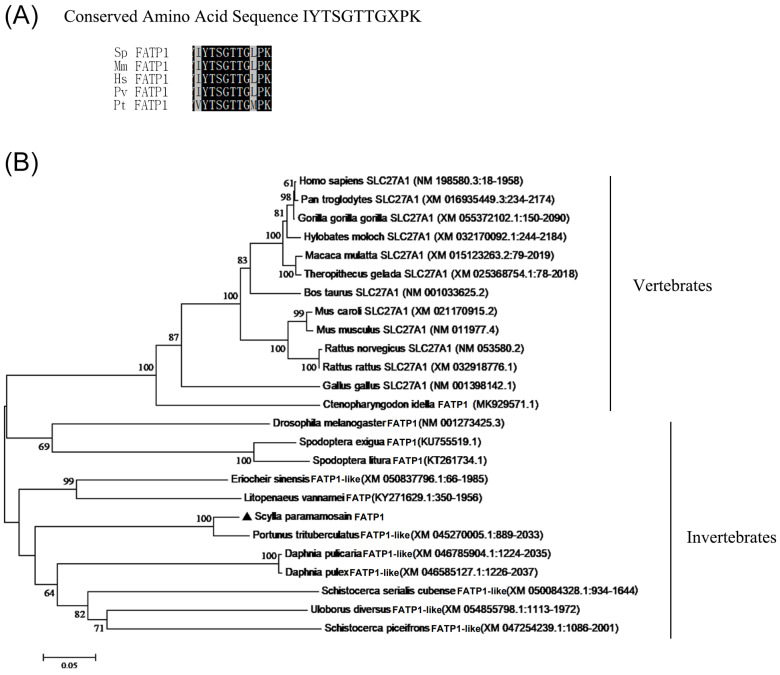
The mud crab FATP1 is a member of the FATP family. (**A**) Conserved amino acid sequence alignment of IYTSGTTGXPK from mud crab FATP1 and its orthologs of other species. (**B**) Phylogenetic tree of FATP1 ortholog amino acid sequences in some vertebrates and invertebrates. The FATP1 orthologs of *Homo sapiens* (NP_940982.1), *Mus musculus* (NP_036107.1), *Penaeus vannamei* (XP_015907328.1) and *Parasteatoda tepidariorum* (KY271629.1/ARO77488.1) were compared with *S. paramamosain* FATP1. Mud crab FATP1 is marked by the black triangle. The neighbor-joining (NJ) polygenetic tree was tested by bootstrapping using 1000 replications.

The full-length cDNA sequence of the FATP1 gene comprised a 1929 base pair open reading frame (ORF), which encodes a polypeptide with 643 amino acids. The nucleotide sequence is accessible in the GenBank database under accession number OR232206. The predicted FATP1 polypeptide has a molecular weight of 71.777 kDa and a theoretical isoelectric point of 8.06. The deduced FATP1 polypeptide exhibits all characteristic features of the FATP family, including a conserved amino acid sequence (IYTSGTTGXPK, as shown in Figure 1A), an acyl-CoA-synthase-related domain (illustrated in Figure S1 [37]) and three predicted transmembrane regions located at amino acid residues 15–43, 134–160 and 392–419 within the N-terminus. Homologous sequence alignment indicated that the FATP1 of *S. paramamosain* shares a low sequence identity of about 45% with vertebrates and a relatively high identity of about 64% with invertebrates, particularly with *Penaeus vannamei* (about 65%) (Figure S2). Phylogenetic analysis reveals that it clusters with other FATP1 orthologs from decapod crustaceans, notably *Portunus trituberculatus*, and is distinctly separated from the FATP1 orthologs of vertebrates (Figure 1B). 

### 3.2. Tissue Distribution Pattern of FATP1 in Mud Crabs

**Figure 2:**
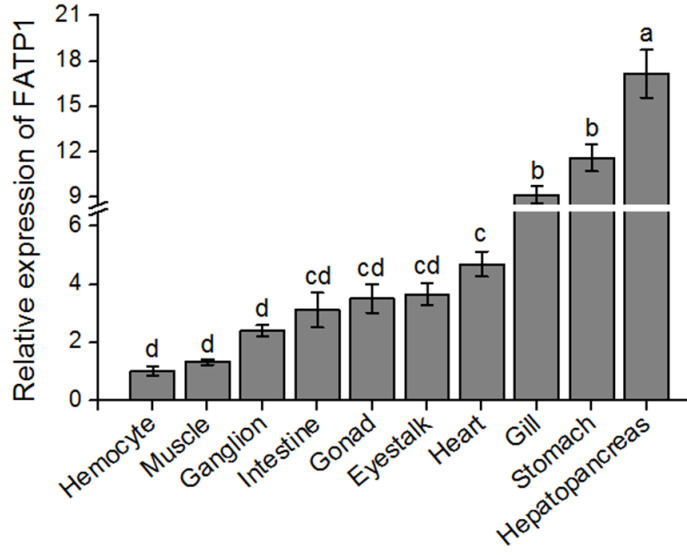
Relative tissue distribution profile of *FATP1* in mud crab determined by qPCR. Values are means ± SEM (n = 6) as fold change from the hemocyte and bars with different superscript letters indicate significant differences among the detected tissues (*p* < 0.05; ANOVA, Tukey’s test).

The *FATP1* gene exhibited diverse expression patterns across various tissues of the mud crab, with the hepatopancreas demonstrating the highest expression level (*p* < 0.05), followed by stomach, gill, heart, eyestalk, gonad and intestine (Figure 2). Comparatively lower expression levels of *FATP1* were observed in ganglion, muscle and hemocyte, with hemocyte showing the lowest expression level among all tissues examined.

### 3.3. Expression of FATP1 in Hepatopancreas of Crabs Fed Different Dietary n-3 LC-PUFA Levels

**Figure 3:**
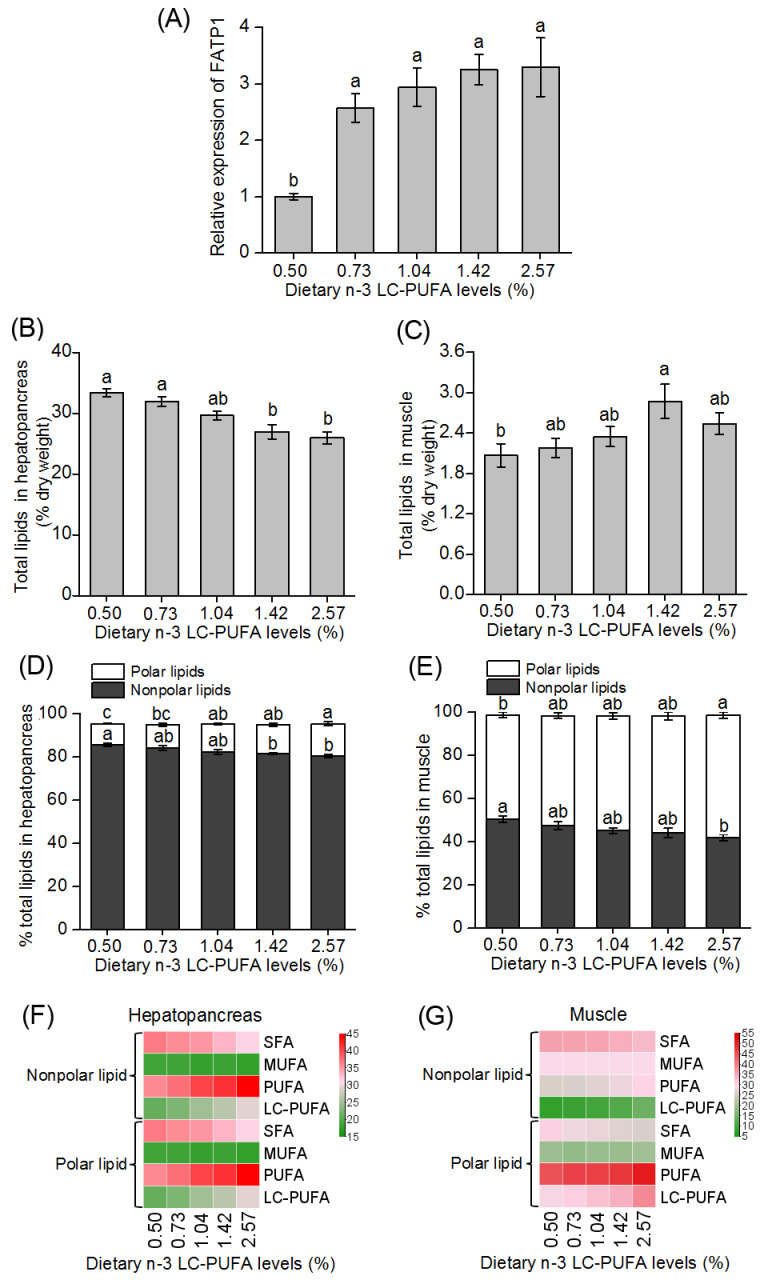
The mud crab FATP1 may be involved in long-chain polyunsaturated fatty acid (LC-PUFA) metabolism and deposition. (**A**) The relative mRNA expression levels of *FATP1* in hepatopancreas of mud crabs fed different dietary levels of n-3 LC-PUFA. Total lipid contents (% dry weight) of (**B**) hepatopancreas and (**C**) muscle of mud crabs fed different dietary levels of n-3 LC-PUFA. Polar and nonpolar lipid contents (% total lipid) of (**D**) hepatopancreas and (**E**) muscle of mud crabs fed different dietary levels of n-3 LC-PUFA. The heatmap visualization of the abundance of total saturated fatty acid (SFA), monounsaturated fatty acid (MUFA), polyunsaturated fatty acid (PUFA) and LC-PUFA in polar and nonpolar lipids of (**F**) hepatopancreas and (**G**) muscle of mud crabs fed different dietary levels of n-3 LC-PUFA. Before analysis, all data were checked for homogeneity. Data are presented as means ± SEM (n = 3). Bars with different superscript letters in panels (**A**–**E**) indicate significant differences.

The expression level of the *FATP1* mRNA in hepatopancreas increased significantly as dietary levels of n-3 LC-PUFA increased. Crabs fed the diets with 0.73, 1.04, 1.42 and 2.57% n-3 LC-PUFA levels showed significantly higher expression levels of the *FATP1* than crabs fed the diet with the lowest (0.50%) level of n-3 LC-PUFA (*p* < 0.05) (Figure 3A).

### 3.4. Total Lipid and FA Composition in Hepatopancreas and Muscle of Crabs Fed Different Dietary n-3 LC-PUFA Levels

The total lipid content in hepatopancreas of crabs significantly decreased with increasing dietary levels of n-3 LC-PUFA (Figure 3B), while the opposite pattern was observed for total lipid content of muscle (*p* < 0.05) (Figure 3C). Among lipids, PLs (mainly of phosphatidylcholine and phosphatidylethanolamine) in hepatopancreas and muscle increased significantly as the dietary level of n-3 LC-PUFA increased, while the NLs (consisting primarily of triacylglycerols) decreased significantly (*p* < 0.05) (Figure 3D,E). Moreover, the LC-PUFA contents, especially EPA and DHA (see Supplementary Tables S3 and S4), in both PLs and NLs in hepatopancreas (Figure 3F) and muscle (Figure 3G) increased significantly with increased dietary level of n-3 LC-PUFA (*p* < 0.05).

### 3.5. Total Lipid, TG and T-CHO Contents and FA Compositions in Hepatopancreas and Muscle after Knockdown of the FATP1 Gene in Mud Crab

**Figure 4:**
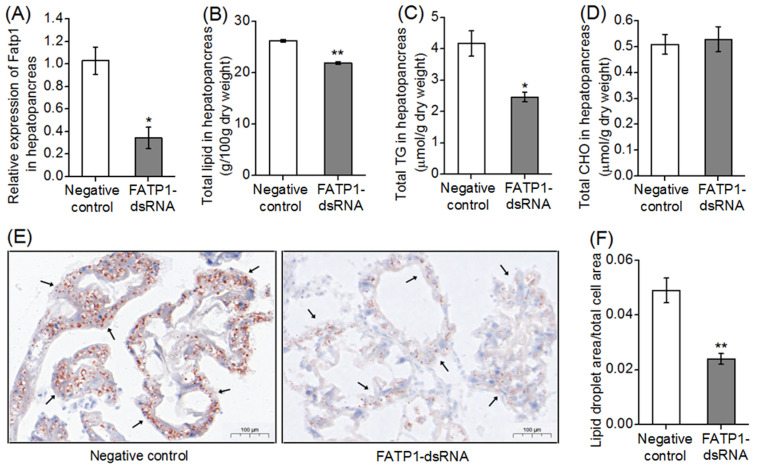
The mud crab FATP1 was involved in lipid metabolism and deposition in hepatopancreas. (**A**) Relative mRNA expression levels of *FATP1* in hepatopancreas. (**B**) Total lipid (g/100g dry weight), (**C**) triacylglycerols (μmol/g dry weight) and (**D**) total cholesterol contents (μmol/g dry weight) of hepatopancreas of mud crab after knockdown of *FATP1* with continuous dsRNA injection. (**E**) Mud crab hepatopancreas stained with Oil Red O after knockdown of *FATP1*, and (**F**) lipid droplet content (area/total cell area) of mud crab hepatopancreas stained with ORO after knockdown of *FATP1*. Crabs treated with PBS only were considered as the negative control. Black arrows indicate lipid droplets stained with ORO. Data are presented as means ± SEM (n = 4) as fold change from the negative controls. *, *p* < 0.05; **, *p* < 0.01 vs. the negative control.

After 2 weeks of continuous dsRNA injection, the expression level of *FATP1* in the hepatopancreas decreased significantly compared with the control group (Figure 4A). In the hepatopancreas of crabs after knockdown of *FATP1*, the concentration of TG and the contents of total lipid were reduced significantly (*p* < 0.05) (Figure 4B,C), while no difference was found in the content of total cholesterol (Figure 4D) compared with the control group. Significantly fewer lipid droplets were observed in crabs treated with *FATP1* dsRNA than in crabs of the control group (*p* < 0.01) as evident in hepatopancreas stained with ORO (Figure 4E,F).

No significant differences were found in the FA compositions of hepatopancreas between crabs treated with *FATP1* dsRNA and the negative control, other than higher levels of 20:1n-9 and total monounsaturated fatty acids (MUFAs, fatty acids containing one double bond) and a lower level of 12:0 (Table 1). Total saturated fatty acids (SFAs, fatty acids excluding a double bond) and total contents of LC-PUFA and n-3 LC-PUFA in the hepatopancreas were slightly, but not significantly, decreased when compared with the control group. Similarly, significantly increased MUFA contents and a nonsignificant trend for lower total contents of LC-PUFA, n-6 LC-PUFA and n-3 LC-PUFA were also observed in muscle of crabs treated with *FATP1* dsRNA (Table 1). However, compared with the control group, no difference was found in total SFA contents in muscle of crabs after treatment with *FATP1* dsRNA, although the contents of 12:0 and 14:0 decreased significantly (*p* < 0.05). 

### 3.6. Expression of Lipid-Metabolism-Related Genes in Hepatopancreas of Crabs after Knockdown of the FATP1 Gene

**Figure 5:**
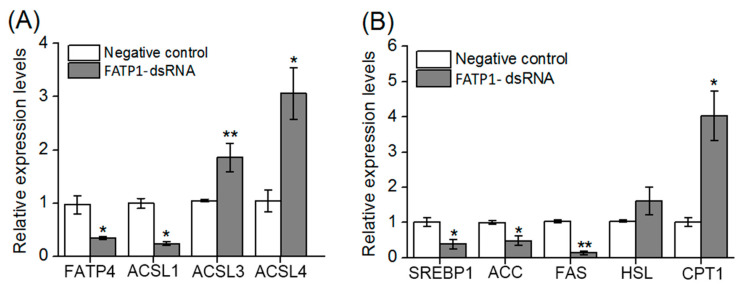
Knockdown of *FATP1* affected the expression levels of genes related to fatty acid uptake, transportation, oxidation and synthesis. (**A**) Expression levels of genes related to fatty acid uptake and transportation, and (**B**) synthesis and oxidation in hepatopancreas of mud crab after *FATP1* knockdown were determined by qPCR. Data are presented as means ± SEM (n = 4). *, *p* < 0.05, **, *p* < 0.01 vs. the negative control.

Compared to the negative control, the expression levels of *FATP4*, *ACSl1*, *SREBP1*, *ACC* and *FAS* were decreased significantly (*p* < 0.05), while the expression levels of *ACSl3*, *ACSL4* and *CPT1* genes were increased significantly in crabs after knockdown of *FATP1* by RNA interference (*p* < 0.05) (Figure 5). However, there were no statistical changes observed in the transcript level of *HSL* in the hepatopancreas of crabs subjected to *FATP1* dsRNA (Figure 5B).

## 4. Discussion

The FATP proteins are a family of membrane-bound proteins that contribute to the selective cellular absorption and activation of LCFA in mammals [1,2]. Among the members of the FATP family, FATP1 is involved in the process of LCFA transportation, oxidation, triacylglycerol synthesis, lipid accumulation and adipocyte differentiation in tissues with rapid FA metabolism, such as the heart, skeletal muscle, liver and adipose tissue [2,9,11,13]. In vertebrates, especially mammals, the role of FATP1 in lipid metabolism has been studied extensively, yet in invertebrates such as crustaceans, it is considerably less known. In this study, we cloned the cDNA of a FATP1 gene from a crustacean species, mud crab *S. paramamosain*, and showed it to encode a putative FATP1 protein of 643 amino acids, similar to human FATP1 protein that contains 646 amino acids [4,9]. The deduced putative FATP1 polypeptide of mud crab contained several common representative characteristics of the FATP family, including a conserved amino acid sequence IYTSGTTGXPK, an acyl-CoA-synthase-related domain and three predicted transmembrane segments [2,4,37]. Bioinformatic analysis result showed that mud crab FATP1 shared relatively high sequence identity with FATP orthologs of other crustaceans, which proved the identity of the *S. paramamosain* FATP1 as a member of the FATP family.

A previous study in invertebrates demonstrated that a FATP homologue cloned from *Eilema japonica* was expressed predominantly in the pheromone gland, and its expression in *E. coli* enhanced the uptake of LCFA (C18 and C20) but not pheromone precursor hydrocarbons [38]. In the present study, the *S. paramamosain FATP1* gene was highly expressed in the hepatopancreas, stomach, gill and heart, which is different to the tissue distribution pattern in vertebrates [9,11], where the *FATP1* gene was highly expressed in tissues with high activity of FA consumption and oxidation such as the heart and skeletal muscle, as well as the liver and adipose tissue. The significantly highest expression of *FATP1* in the hepatopancreas of crustaceans, which is the primary organ responsible for lipid metabolism and energy storage in these organisms, suggests its potential role in lipid absorption and storage [39,40]. This finding further substantiates the classification of FATP1 in mud crabs as an ortholog of FATP1.

In different mammalian cell types and tissues, there is ongoing debate regarding whether FATP1 promotes or inhibits the esterification and oxidation of FAs [13]. In this study, we found that both TG and total lipid contents of hepatopancreas were reduced, while there is no change in the content of CHO in crabs after knockdown of *FATP1*, suggesting that the *FATP1* gene promoted lipid uptake and accretion in the hepatopancreas of mud crabs. This was consistent with previous studies that showed that the overexpression of *FATP1* promoted preadipocyte differentiation and fat deposition in bovine adipocytes, while the interference of *FATP1* exerted an opposing effect on adipocyte differentiation [14,41]. The mammal FATP4 is the transport protein most closely related to ATP1 and is highly expressed in skeletal muscle, heart and adipose tissue [42]. A previous study found that the loss of *FATP1* in 3T3-L1 cell lines did not affect the expression of *FATP4* but led to reduced triacylglycerol deposition in these cells [14]. It seems like the loss of key *FATP1* functions would not affect the expression of *FATP4*. However, in the present study, knockdown of *FATP1* reduced the expression of *FATP4*. This implies that the expression of genes involved in lipid metabolism like *FATP4* would be more likely to be affected by the loss of key functions of *FATP1*. Moreover, the expression levels of *ACSL* genes, which encode proteins that activate exogenous or endogenous FAs to enter metabolic pathways [43,44,45], were also impacted by *FATP1* with *ACSL3* expression being decreased, similar to *FATP4*, in the hepatopancreas of crabs after knockdown of *FATP1*. This suggested that the mud crab *FATP1* may affect the transport and partitioning of FAs, thus regulating hepatopancreatic lipid accumulation. Furthermore, the expression levels of genes related to lipogenesis, including *ACC*, *FAS* and *SREBP1*, all decreased in the hepatopancreas of crabs subjected to knockdown of *FATP1* in the present study. ACC is the rate-limiting enzyme involved in de novo fatty acid synthesis by catalyzing the ATP-dependent carboxylation of acetyl-CoA to malonyl-CoA [44], while SREBP1 is a key transcription factor regulating several genes of lipid biosynthesis [46]. Thus, knockdown of *FATP1* gene expression was associated with a general down-regulation of lipogenesis in the hepatopancreas, suggesting that *FATP1* and its function were important for the stimulation of lipogenesis. Conversely, the expression level of *CPT1*, which is related to lipid catabolism and FA β-oxidation [47], was increased in the hepatopancreas of crabs subjected to *FATP1* knockdown. This suggested that *FATP1* plays an important role in inhibiting lipid catabolism. It appears unlikely that these effects on the expression of other genes involved in lipid metabolism could be a direct effect of the gene knockdown and would more likely be a result of the loss of key functions of *FATP1*. However, the precise mechanism of the effects of *FATP1* knockdown on the expression of other genes related to lipid metabolism currently remains unclear and requires further study. Interestingly, in this context, the expression levels of *ACSL3* and *ACSL4*, which have substrate preferences for LC-PUFA, like EPA and ARA [40,41], were also increased in the hepatopancreas of crabs subjected to *FATP1* knockdown. This observation contrasts with the FA composition of the hepatopancreas, which demonstrated a tendency to exhibit reduced levels of n-3 LC-PUFA in crabs subjected to *FATP1* knockdown. Therefore, the interaction between the roles of *FATP1* and *ACSLs* and the differential impact of *FATP1* knockdown on the expression of different members of the *ACSL* family also requires further investigation.

The FATP family of membrane-associated proteins has been suggested to be an important mediator of LCFA trafficking into cells [48]. The use of stable isotopically labeled FAs demonstrated that FATP2a was involved in the uptake and activation of exogenous FAs, with a preference toward n-3 PUFA (18:3-3 and 22:6n-3) as well as very long-chain saturated fatty acids (24:0) [49]. The application of small interfering RNA (siRNA) to inhibit the expression of *FATP4* in Neuro2a cells resulted in a reduction in the activation of both long-chain (16:0) and very-long-chain saturated fatty acids (24:0), while overexpression of *FATP4* in COS-1 cells resulted in enhanced uptake of 16:0 [50]. It was also shown that *FATP1* played a vital role in insulin-stimulated LCFA absorption and activation. Specifically, insulin-stimulated FA uptake was completely abolished in *FATP1*-null adipocytes and greatly reduced in skeletal muscle of *FATP1*-knockout animals [16]. In terms of FA composition, there was specifically a reduction in total LC-PUFA contents in both the muscle and hepatopancreas of crabs following treatment with *FATP1* dsRNA. These findings suggest that the FATP1 protein in mud crabs may exhibit a substrate preference for LC-PUFA. Furthermore, it was also found that increasing dietary levels of n-3 LC-PUFA significantly up-regulated the expression of *FATP1* in the hepatopancreas of mud crabs, which corresponded with increased levels of LC-PUFA in PLs and NLs in hepatopancreas and muscle tissues. Similarly, in mammals, overexpression of *FATP1* in rats and mice leads to an increase in the rate of LCFA transportation and both systemic deposition of FAs and intramuscular lipid accumulation [19,51,52]. Overall, these results suggested *FATP1* plays a key role in uptake and esterification of LC-PUFA in hepatopancreas and muscle tissues of mud crabs.

## 5. Conclusions

In conclusion, we successfully cloned and characterized a novel *FATP1* gene from mud crab *S. paramamosain*, a species of decapod crustacean. Beside possessing all the typical features of the FATP family, the mud crab FATP1 shared high homology with other FATP orthologs identified from crustaceans. The expression of *FATP1* was widely detected in tissues, particularly in the more metabolically active tissues such as the hepatopancreas, stomach and gill. Decreased lipid accumulation, as well as reduced expression levels of genes related to FA uptake, transportation, oxidation and synthesis, was found in the hepatopancreas of crabs subjected to knockdown of *FATP1* in vivo. Crabs fed higher dietary levels of n-3 LC-PUFA showed increased *FATP1* gene expression in the hepatopancreas and elevated contents of LC-PUFA, especially EPA and DHA, in both PLs and NLs in the hepatopancreas and muscle. Overall, the present study indicated that FATP1 identified from *S. paramamosain* is involved in LC-PUFA metabolism and deposition in crustaceans, enhancing our understanding of lipid accumulation in invertebrates.

## Figures and Tables

**Table 1:** The main fatty acid compositions (% total fatty acid) of hepatopancreas and muscle of mud crabs after knockdown of *FATP1*.

Fatty Acid	Hepatopancreas			Muscle		
	Negative Control *	*FATP1* dsRNA	*p*-Value	Negative Control	*FATP1* dsRNA	*p*-Value
12:0	3.45 ± 0.20	2.67 ± 0.23	0.044	1.04 ± 0.19	0.40 ± 0.03	0.027
14:0	4.40 ± 0.23	3.92 ± 0.42	0.352	2.24 ± 0.29	1.23 ± 0.04	0.028
16:0	15.97 ± 0.63	17.62 ± 1.88	0.436	17.83 ± 0.33	18.88 ± 0.81	0.296
18:0	5.63 ± 0.30	3.73 ± 0.94	0.103	10.89 ± 0.25	11.45 ± 0.43	0.319
16:1n-9	3.52 ± 0.28	4.24 ± 0.80	0.427	2.20 ± 0.26	2.26 ± 0.35	0.894
16:1n-7	0.67 ± 0.07	1.52 ± 0.37	0.066	0.89 ± 0.19	4.89 ± 1.17	0.028
18:1n-9	14.34 ± 0.31	14.70 ± 0.32	0.453	11.00 ± 0.54	10.39 ± 0.80	0.558
20:1n-9	0.85 ± 0.10	1.85 ± 0.34	0.030	0.55 ± 0.12	0.91 ± 0.06	0.054
18:2n-6	13.12 ± 0.58	13.28 ± 0.74	0.870	7.19 ± 0.74	6.50 ± 0.62	0.509
18:3n-3	4.32 ± 0.12	3.87 ± 0.14	0.051	1.59 ± 0.18	1.44 ± 0.28	0.679
20:4n-6	6.94 ± 0.24	6.68 ± 0.48	0.649	12.48 ± 0.94	10.81 ± 0.31	0.168
22:4n-6	0.45 ± 0.12	0.44 ± 0.03	0.948	1.06 ± 0.20	1.03 ± 0.23	0.919
20:5n-3	6.58 ± 0.27	6.08 ± 0.46	0.385	14.22 ± 0.19	11.97 ± 1.31	0.165
22:5n-3	1.40 ± 0.08	1.42 ± 0.06	0.866	1.35 ± 0.06	0.98 ± 0.14	0.071
22:6n-3	6.41 ± 0.34	6.27 ± 0.42	0.806	7.18 ± 0.40	5.98 ± 0.85	0.268
ΣSFA ^1^	29.44 ± 1.32	27.94 ± 1.60	0.496	31.99 ± 0.68	31.96 ± 1.29	0.985
ΣMUFA ^2^	19.39 ± 0.49	22.31 ± 1.04	0.044	14.64 ± 0.69	18.45 ± 0.95	0.032
ΣPUFA ^3^	39.21 ± 1.54	38.04 ± 1.59	0.615	45.07 ± 1.02	38.70 ± 3.14	0.126
ΣLC-PUFA ^4^	21.78 ± 0.85	20.89 ± 1.27	0.584	36.28 ± 0.70	30.76 ± 2.57	0.107
Σn-3 LC-PUFA	14.38 ± 0.55	13.77 ± 0.81	0.628	22.75 ± 0.60	18.92 ± 2.24	0.174
Σn-6 LC-PUFA	7.36 ± 0.29	7.12 ± 0.50	0.701	13.53 ± 1.13	11.84 ± 0.43	0.233

* Crabs treated with PBS only were considered as the negative control. Data are presented as means ± SEM (n = 4). ^1^ SFA, saturated fatty acid. ^2^ MUFA, monounsaturated fatty acid. ^3^ PUFA, polyunsaturated fatty acid. ^4^ LC-PUFA, long-chain polyunsaturated fatty acid, includes 20:4n-6, 22:4n-6, 20:5n-3, 22:5n-3 and 22:6n-3.

## Data Availability

The data presented in this study are available on request from the corresponding author. The availability of the data is restricted to investigators based at academic institutions.

## Supplementary Materials

The following supporting information can be downloaded at: https://www.mdpi.com/article/10.3390/ani14202969/s1, Table S1: Ingredients and proximate composition of the experimental diets (% of dry weight). Table S2: Primers used in this study. Table S3: Fatty acid composition of the muscle in crabs fed different dietary n-3 LC-PUFA levels. Table S4: Fatty acid composition of the hepatopancreases in crabs fed different dietary n-3 LC-PUFA levels. Figure S1. Open reading frame sequence of *FATP1* in *S. paramamosain* and its predicted amino acid sequence. Figure S2. Multiple alignment of FATP1 homologues in different species.
