# Supplementary material for: Identification and Functional Characterization of the FATP1 Gene from Mud Crab, Scylla paramamosain
Source: Animals (Basel). 2024 Oct 15;14(20):2969. doi: 10.3390/ani14202969 (PMC11506284; doi:10.3390/ani14202969)
Supplement: Supplementary file 1 [file animals-14-02969-s001.zip › animals-3213456_supplementary file.pdf]

Table S1. Ingredients and proximate composition of the experimental diets (% of dry weight)

	Dietary n-3 LC-PUFA levels (% of dry weight)				
	0.50	0.73	1.04	1.42	2.57
Ingredients					
Vegetable oil[1]	10.00	10.00	10.00	10.00	10.00
Fish oil	5.00	5.00	5.00	5.00	5.00
Coconut oil	29.50	25.50	20.00	15.00	0.00
EPA-enriched oil[2]	0.00	3.00	7.00	11.00	22.60
DHA-enriched oil[3]	1.50	2.50	4.00	5.20	9.00
ARA-enriched oil[4]	10.00	10.00	10.00	9.80	9.40
Others[5]	944.00	944.00	944.00	944.00	944.00
Proximate composition (% of dry weight)					
Moisture	7.77	7.66	7.69	7.60	7.79
Crude protein	41.97	41.07	41.12	41.80	41.91
Crude lipid	7.79	7.78	7.85	7.79	7.80
n-3 LC-PUFA	0.50	0.73	1.04	1.42	2.57
n-6 LC-PUFA	0.51	0.51	0.53	0.53	0.51
Dietary fatty acid composition (% total fatty acids)					
12:0	15.92	14.09	7.95	6.91	0.00
14:0	8.08	7.30	5.50	4.88	1.85
16:0	15.54	15.92	15.41	14.38	12.44
16:1n-9	1.82	1.90	1.89	1.90	1.83
18:0	5.60	5.65	5.76	5.07	4.18
18:1n-9	13.99	13.63	14.34	13.36	10.96
18:2n-6	16.07	15.99	15.81	16.08	15.18
18:3n-3	6.81	6.78	6.28	6.78	6.64
20:4n-6 (ARA)	6.56	6.54	6.69	6.78	6.60
20:5n-3 (EPA)	2.99	4.43	5.77	8.39	15.25
22:5n-3 (DPA)	0.49	0.74	1.22	1.56	2.56
22:6n-3 (DHA)	3.41	5.01	7.48	9.89	17.64
ΣSFA[6]	46.98	44.77	36.10	32.35	19.06
ΣMUFA[7]	15.81	15.53	16.23	15.26	12.79
ΣPUFA[8]	36.33	39.49	43.24	49.47	63.87
Σn-6 PUFA	22.63	22.53	22.50	22.87	21.78
Σn-3 PUFA	13.70	16.96	20.74	26.61	42.10
Σn-3 LC-PUFA[9]	6.89	10.18	14.46	19.84	35.46

[1] Vegetable oil mixture: the ratio of perilla oil to safflower oil is 2.0.

[2] EPA-enriched oil: 31.41% DHA, 57.24% EPA and other fatty acids, Xi'an Sheng he Biological Technology Co., Ltd., China.

[3] DHA-enriched oil: 78.89% DHA,15.64% DPA and other fatty acids, Xi'an Sheng he Biological Technology Co., Ltd., China.

[4] ARA-enriched oil: 57.72% ARA, 11.13% 18:0, 8.13% 18:1n-9 and other fatty acids, Xi'an Sheng he Biological Technology Co., Ltd., China.

[5] Included casein (300 mg g^{-1}), soybean protein concentrate (180 mg g^{-1}), α-starch (144 mg g^{-1}), semi skimmed fish meal (100 mg g^{-1}), krill meal (50 mg g^{-1}), yeast extract (50 mg g^{-1}), carboxymethyl cellulose (30 mg g^{-1}), soybean lecithin (20 mg g^{-1}), vitamin and mineral premixes (20 and 20 mg g^{-1}, respectively), monocalcium phosphate (15 mg g^{-1}), cholesterol (8 mg g^{-1}) and choline chloride (7 mg g^{-1}). The vitamin and mineral premixes were obtained from Guangzhou Hinter Bio-technology Co., Ltd., China.

[6] SFA: Saturated fatty acids.

[7] MUFA: Monounsaturated fatty acids.

[8] PUFA: Polyunsaturated fatty acids.

[9] LC-PUFA: Highly unsaturated fatty acids.

Table S2. Primers used in this study.

Name of primer	Sequence of primers (5′→3′)	Application	Reference
5' GSP	TGGCATACCAAAGGACACTGGTCACC	5′ RACE	
3' GSP	CTCTTCCCAGCTGTCTATCCTGTGGC	3′ RACE	
LITMUS-Fatp1-F	GGAAGATCTCCTTGGAGGTTCTTTAAC	Vector Construction	
LITMUS-Fatp1-R	GAAAGGCCTAACTTCAGCACCACACAC		
Q-Sp-18S-F	CAGACAAATCGCTCCACCAAC	qRT-PCR	[30]
Q-Sp-18S-R	GACTCAACACGGGGAACCTCA		
Q-Sp-Acsl1-F	ATCAACCAGGCTTCCATCAC	qRT-PCR	[30]
Q-Sp-Acsl1-R	CATATGGTGCACAGGTCGTC		
Q-Sp-Acsl3-F	ACCAGCTCAAAGAGACTCCC	qRT-PCR	[30]
Q-Sp-Acsl3-R	CCAATGACGCCACTGACAAG		
Q-Sp-Acsl4-F	CACCGTATCACCGTATGCAC	qRT-PCR	[30]
Q-Sp-Acsl4-R	ATCCTCCATTACCACCACCG		
Q-Sp-Fatp1-F	GCGTGTGGATTTGTGTCAGT	qRT-PCR	[30]
Q-Sp-Fatp1-R	GCCTTCTTGTCTGCATAGCC		
Q-Sp-Fatp4-F	CCTCCTCAACATGCCAGAGA	qRT-PCR	[30]
Q-Sp-Fatp4-R	GAGCCACAGGATAGACAGCT		
Q-Sp-Srebp1-F	TGCCTGGCCGTACTATACTG	qRT-PCR	[30]
Q-Sp-Srebp1-R	ACTGCCCCTTAGACAAGTCC		
Q-Sp-Acc-F	GTAAGCCGAGTCAGGAAACCAC	qRT-PCR	[30]
Q-Sp-Acc-R	GACGACCCAACCCTAACAACC		
Q-Sp-Fas-F	ACTCTGGAACGAGAGGCTGA	qRT-PCR	[30]

Q-Sp-Fas-R	ATGTTGGAGTCAGGGAGCAC

Table S3. Fatty acid composition of the muscle in crabs fed different dietary n-3 LC-PUFA levels.

Fatty acid	Dietary n-3 LC-PUFA levels				
	0.50	0.73	1.04	1.42	2.57
Polar lipids					
12:0	0.55 ± 0.02[b]	0.57 ± 0.02[b]	0.51 ± 0.01[ab]	0.53 ± 0.003[ab]	0.47 ± 0.03[a]
14:0	0.81 ± 0.01[ab]	0.82 ± 0.01[b]	0.80 ± 0.01[ab]	0.72 ± 0.04[ab]	0.63 ± 0.09[a]
16:0	16.49 ± 0.04	16.52 ± 0.09	16.49 ± 0.10	16.15 ± 0.18	16.18 ± 0.10
18:0	12.73 ± 0.10[c]	10.21 ± 0.68[b]	9.83 ± 0.35[ab]	9.00 ± 0.35[ab]	8.51 ± 0.28[a]
16:1n-7	3.03 ± 0.03[b]	2.67 ± 0.12[ab]	2.72 ± 0.07[ab]	2.58 ± 0.18[a]	2.80 ± 0.04[ab]
18:1n-9	10.90 ± 0.25	10.81 ± 0.13	10.58 ± 0.10	11.01 ± 0.05	11.35 ± 0.38
20:1n-9	5.24 ± 0.09[ab]	4.89 ± 0.16[a]	5.62 ± 0.04[b]	5.56 ± 0.14[ab]	5.62 ± 0.28[b]
18:2n-6	13.84 ± 0.32[c]	13.52 ± 0.08[c]	11.97 ± 0.26[b]	11.24 ± 0.05[b]	10.29 ± 0.06[a]
18:3n-3	2.36 ± 0.02	2.32 ± 0.02	2.32 ± 0.07	2.32 ± 0.03	2.19 ± 0.07
20:4n-6	4.09 ± 0.08	4.13 ± 0.08	4.11 ± 0.07	4.06 ± 0.02	4.20 ± 0.09
20:5n-3	14.95 ± 0.24[a]	15.85 ± 0.26[ab]	16.51 ± 0.26[bc]	17.24 ± 0.26[c]	18.53 ± 0.33[d]
22:5n-3	0.41 ± 0.17	0.49 ± 0.20	0.56 ± 0.23	0.73 ± 0.30	0.83 ± 0.34
22:6n-3	9.15 ± 0.40[a]	10.24 ± 0.24[ab]	11.09 ± 0.15[bc]	12.10 ± 0.39[c]	14.95 ± 0.04[d]
ΣSFA [1]	30.57 ± 0.10[c]	28.12 ± 0.59[b]	27.63 ± 0.40[ab]	26.39 ± 0.56[ab]	25.79 ± 0.48[a]
ΣMUFA[2]	19.16 ± 0.27	18.36 ± 0.18	18.93 ± 0.06	19.14 ± 0.02	19.76 ± 0.69
ΣPUFA [3]	44.80 ± 0.83[a]	46.55 ± 0.36[ab]	46.56 ± 0.65[ab]	47.69 ± 0.48[b]	50.99 ± 0.26[c]
ΣLC-PUFA[4]	28.59 ± 0.56[a]	30.71 ± 0.44[b]	32.28 ± 0.34[b]	34.13 ± 0.46[c]	38.51 ± 0.25[d]
Nonpolar lipids					
12:0	1.73 ± 0.07[ab]	1.93 ± 0.06[b]	1.86 ± 0.04[ab]	1.42 ± 0.12[a]	1.60 ± 0.21[ab]
14:0	1.92 ± 0.22	1.68 ± 0.31	1.19 ± 0.04	1.23 ± 0.08	1.28 ± 0.10

16:0	20.00 ± 0.70	19.26 ± 0.79	19.28 ± 1.13	18.66 ± 1.44	18.10 ± 1.57
18:0	12.31 ± 0.34	13.01 ± 0.75	13.16 ± 0.20	13.01 ± 0.75	12.03 ± 0.66
20:0	3.45 ± 0.20	3.45 ± 0.20	3.45 ± 0.20	3.45 ± 0.20	3.45 ± 0.20
16:1n-7	2.38 ± 0.05	2.17 ± 0.15	2.09 ± 0.13	2.02 ± 0.02	2.12 ± 0.12
18:1n-9	26.40 ± 0.34	26.98 ± 0.46	26.45 ± 0.54	26.70 ± 0.56	26.52 ± 0.48
20:1n-9	0.58 ± 0.003	0.61 ± 0.01	0.60 ± 0.01	0.60 ± 0.01	0.61 ± 0.02
18:2n-6	12.40 ± 0.39[a]	12.01 ± 0.32[ab]	11.86 ± 0.13[ab]	11.64 ± 0.03[ab]	11.29 ± 0.07[b]
18:3n-3	5.19 ± 0.05	5.03 ± 0.21	4.81 ± 0.41	4.75 ± 0.55	4.56 ± 0.47
20:4n-6	1.88 ± 0.04[a]	1.74 ± 0.01[b]	1.72 ± 0.02[b]	1.81 ± 0.04[ab]	1.82 ± 0.04[ab]
20:5n-3	3.26 ± 0.04[a]	3.69 ± 0.07[b]	4.43 ± 0.02[c]	5.39 ± 0.02[d]	6.86 ± 0.02[e]
22:6n-3	2.95 ± 0.01[a]	3.29 ± 0.03[b]	3.75 ± 0.01[c]	4.34 ± 0.02[d]	5.66 ± 0.11[e]
ΣSFA	35.96 ± 0.66[a]	35.88 ± 0.36[a]	35.49 ± 0.42[a]	34.32 ± 0.50[ab]	33.02 ± 0.62[b]
ΣMUFA	29.36 ± 0.30	29.76 ± 0.36	29.14 ± 0.42	29.32 ± 0.56	29.25 ± 0.60
ΣPUFA	25.69 ± 0.35[a]	25.77 ± 0.21[a]	26.57 ± 0.28[ab]	27.93 ± 0.58[b]	30.20 ± 0.35[c]
ΣLC-PUFA	8.10 ± 0.01[a]	8.72 ± 0.10[b]	9.90 ± 0.03[c]	11.54 ± 0.02[d]	14.34 ± 0.11[e]

Data are presented as means ± SEM (n = 3).

[1] SFA, saturated fatty acids. [2] MUFA, monounsaturated fatty acids. [3] PUFA, polyunsaturated fatty acids.

[4] LC-PUFA, long-chain polyunsaturated fatty acids.

Table S4. Fatty acid composition of the hepatopancreases in crabs fed different dietary n-3 LC-PUFA levels.

Fatty acid	Dietary n-3 LC-PUFA levels				
	0.50	0.73	1.04	1.42	2.57
Polar lipids					
12:0	1.19 ± 0.12[ab]	1.74 ± 0.43[b]	1.88 ± 0.13[b]	1.21 ± 0.04[ab]	0.62 ± 0.04[a]
14:0	2.14 ± 0.06[a]	2.57 ± 0.37[ab]	3.13 ± 0.17[b]	2.38 ± 0.08[ab]	2.06 ± 0.20[a]
16:0	20.68 ± 0.89[b]	20.01 ± 0.64[ab]	19.60 ± 0.54[ab]	19.21 ± 0.21[ab]	17.90 ± 0.19[a]
18:0	12.44 ± 0.48[b]	11.06 ± 0.23[a]	9.99 ± 0.21[a]	9.84 ± 0.06[a]	10.14 ± 0.35[a]
20:0	0.40 ± 0.02[a]	0.46 ± 0.04[ab]	0.56 ± 0.03[b]	0.57 ± 0.01[b]	0.59 ± 0.05[b]
16:1n-7	3.65 ± 0.19	3.68 ± 0.17	3.25 ± 0.18	3.05 ± 0.05	3.33 ± 0.36
18:1n-9	14.40 ± 0.34	14.08 ± 0.59	13.86 ± 0.54	14.53 ± 0.43	13.47 ± 0.23
20:1n-9	0.50 ± 0.09	0.69 ± 0.05	0.68 ± 0.08	0.63 ± 0.16	0.81 ± 0.62
18:2n-6	11.21 ± 0.23	11.53 ± 0.12	11.91 ± 0.06	11.51 ± 0.25	11.16 ± 0.34
18:3n-3	3.72 ± 0.15	3.40 ± 0.13	3.42 ± 0.24	3.31 ± 0.30	3.96 ± 0.16
20:4n-6	7.77 ± 0.14	7.26 ± 0.53	7.53 ± 0.31	7.33 ± 0.06	7.48 ± 0.13
20:5n-3	6.79 ± 0.72[a]	7.84 ± 0.67[ab]	8.60 ± 0.55[abc]	9.37 ± 0.45[bc]	10.91 ± 0.28[c]
22:6n-3	6.45 ± 0.37[a]	7.49 ± 0.51[ab]	8.75 ± 0.52[bc]	9.88 ± 0.32[cd]	10.85 ± 0.23[c]
ΣSFA [1]	36.85 ± 1.47[b]	35.83 ± 1.25[b]	35.16 ± 1.05[ab]	33.20 ± 0.26[ab]	31.32 ± 0.49[a]
ΣMUFA [2]	18.54 ± 0.20	18.45 ± 0.59	17.78 ± 0.78	18.20 ± 0.54	17.61 ± 0.42
ΣPUFA [3]	35.94 ± 1.06[a]	37.52 ± 1.65[ab]	40.20 ± 1.63[abc]	41.39 ± 0.37[bc]	44.37 ± 0.75[c]
ΣLC-PUFA [4]	21.01 ± 1.19[a]	22.59 ± 1.70[ab]	24.87 ± 1.37[abc]	26.57 ± 0.80[bc]	29.24 ± 0.52[c]
Nonpolar lipids					
12:0	3.14 ± 0.16[c]	3.03 ± 0.20[c]	2.74 ± 0.30[c]	1.76 ± 0.07[b]	0.51 ± 0.05[a]
14:0	4.63 ± 0.14[c]	4.37 ± 0.15[c]	4.27 ± 0.32[c]	3.30 ± 0.11[b]	2.09 ± 0.13[a]
16:0	20.65 ± 0.93	20.24 ± 0.83	20.27 ± 0.65	20.16 ± 0.67	19.93 ± 0.67

18:0	6.82 ± 0.14b^{c}	7.47 ± 0.31^{c}	6.31 ± 0.18^{ab}	6.15 ± 0.21^{ab}	5.65 ± 0.21^{a}
20:0	0.73 ± 0.02^{ab}	0.79 ± 0.03^{b}	0.69 ± 0.01^{a}	0.71 ± 0.03^{ab}	0.67 ± 0.02^{a}
16:1n-7	4.58 ± 0.09^{b}	3.99 ± 0.13^{ab}	3.98 ± 0.28^{ab}	3.56 ± 0.09^{b}	3.65 ± 0.25^{b}
18:1n-9	22.23 ± 1.13	21.70 ± 1.09	21.51 ± 1.47	23.50 ± 0.84	22.32 ± 0.80
20:1n-9	0.95 ± 0.02^{b}	0.56 ± 0.04^{a}	0.85 ± 0.02^{b}	0.87 ± 0.05^{b}	0.85 ± 0.05^{b}
18:2n-6	15.54 ± 0.48	14.95 ± 0.31	14.85 ± 0.29	15.27 ± 0.51	14.57 ± 0.60
18:3n-3	4.28 ± 0.06^{a}	5.07 ± 0.18^{b}	4.25 ± 0.12^{a}	4.24 ± 0.09^{a}	4.17 ± 0.13^{a}
20:4n-6	5.50 ± 0.08^{ab}	5.32 ± 0.11^{a}	5.56 ± 0.24^{ab}	6.16 ± 0.07^{bc}	6.04 ± 0.08^{c}
20:5n-3	3.06 ± 0.05^{a}	3.42 ± 0.08^{a}	3.97 ± 0.12^{b}	4.60 ± 0.13^{c}	5.68 ± 0.03^{d}
22:6n-3	3.14 ± 0.05^{a}	3.48 ± 0.05^{a}	4.04 ± 0.07^{b}	4.63 ± 0.08^{c}	5.51 ± 0.15^{d}
ΣSFA	35.96 ± 1.03^{b}	35.90 ± 0.82^{b}	34.28 ± 1.46^{b}	32.07 ± 0.64^{ab}	28.85 ± 0.69^{a}
ΣMUFA	27.76 ± 1.08	26.25 ± 0.99	26.34 ± 1.21	27.91 ± 0.80	26.83 ± 0.59
ΣPUFA	31.52 ± 0.38^{a}	32.24 ± 0.33^{a}	32.67 ± 0.27^{a}	34.89 ± 0.38^{b}	35.97 ± 0.52^{b}
ΣLC-PUFA	11.70 ± 0.16^{a}	12.23 ± 0.19^{a}	13.56 ± 0.43^{b}	15.39 ± 0.24^{c}	17.23 ± 0.18^{d}

Data are presented as means ± SEM (n = 3).

[1] SFA, saturated fatty acids. [2] MUFA, monounsaturated fatty acids. [3] PUFA, polyunsaturated fatty acids.

[4] LC-PUFA, long-chain polyunsaturated fatty acids.

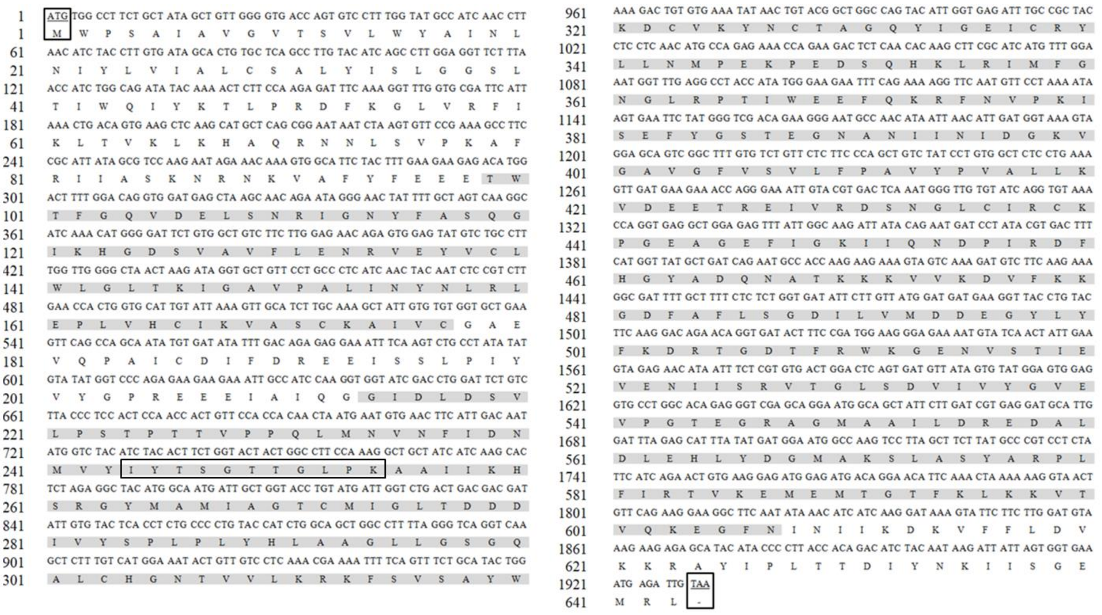

Figure S1. Open reading frame sequence of *FATP1* in *S. paramamosain* and its predicted amino acid sequence. The start codon, termination codon and conserved amino acid sequence IYTSGTTGXPK were marked with boxes. Acyl-CoA synthase related domain was marked in gray.

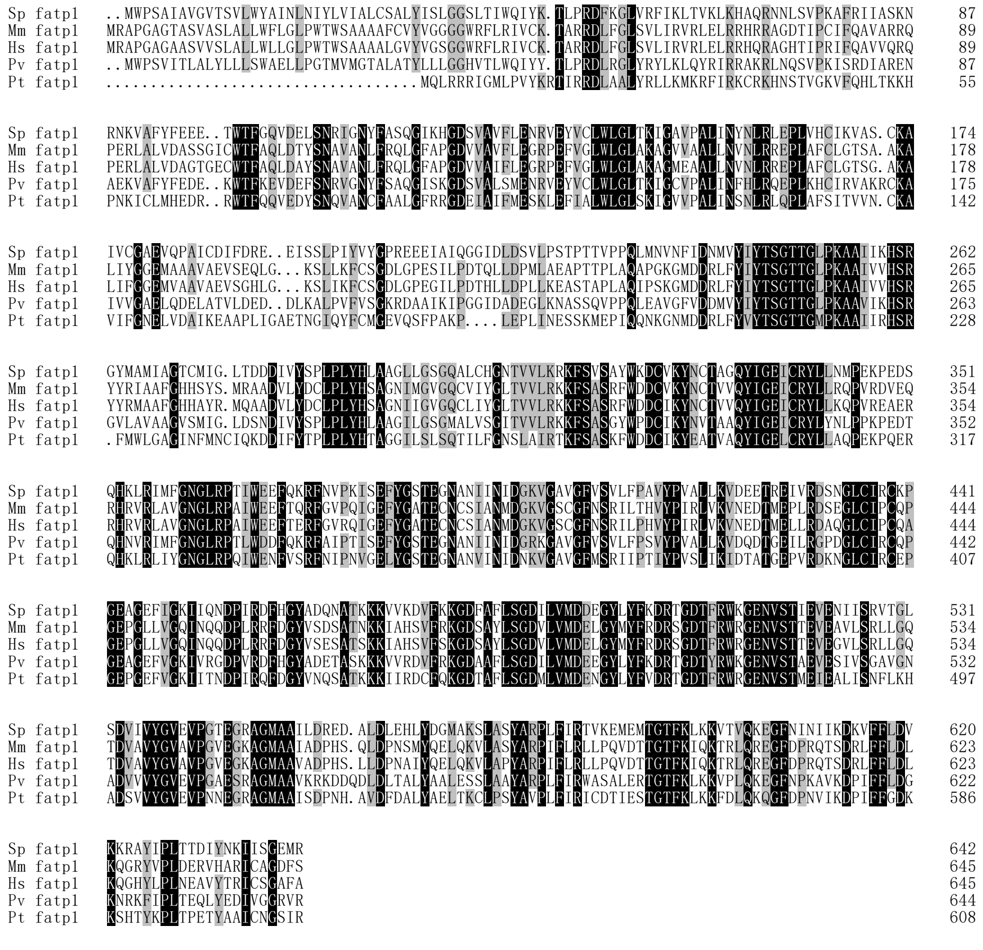

Figure S2. Multiple alignment of FATP1 homologues in different species. FATP1 homologues of *Mus musculus, Homo sapiens, Penaeus vannamei* and *Parasteatoda tepidariorum* were used to multiple

comparison with mud crab FATP1. The consensus sequences were marked out. The relationships between the residues are indicated as follows: non-similar residues, black letters on a white background; block of similarity, white letters on a grey background; identical residues, white letters on a black background.
